# A highly infectious *Plasmodium yoelii* parasite, bearing *Plasmodium falciparum* circumsporozoite protein

**DOI:** 10.1186/s12936-016-1248-z

**Published:** 2016-04-12

**Authors:** Min Zhang, Izumi Kaneko, Tiffany Tsao, Robert Mitchell, Elizabeth H. Nardin, Shiroh Iwanaga, Masao Yuda, Moriya Tsuji

**Affiliations:** HIV and Malaria Vaccine Program, Aaron Diamond AIDS Research Center, Affiliate of The Rockefeller University, New York, NY USA; Department of Pathology, New York University School of Medicine, New York, NY USA; Department of Medical Zoology, Mie University Graduate School of Medicine, Tsu, Mie Japan; Division of Parasitology, Department of Microbiology, New York University School of Medicine, New York, NY USA

**Keywords:** PfCSP/Py, Circumsporozoite protein, *Plasmodium falciparum*, *Plasmodium yoelii*

## Abstract

**Background:**

*Plasmodium* circumsporozoite protein (CSP) is a major surface antigen present in the sporozoite (Spz) stage of a malaria parasite. RTS, S vaccine, the most clinically advanced malaria vaccine, consists of a large portion of *Plasmodium falciparum* CSP (PfCSP). A highly infectious, recombinant rodent malaria, *Plasmodium yoelii* parasite bearing a full-length PfCSP, PfCSP/Py Spz, was needed as a tool to evaluate the role of PfCSP in mediating, protective, anti-malaria immunity in a mouse model.

**Methods:**

A transgenic parasite, PfCSP/Py Spz, was generated by inserting a construct expressing the PfCSP at the locus of the *P. yoelii CSP* gene by double cross-over homologous recombination. Then the biological and protective properties of PfCSP/Py Spz were determined.

**Results:**

This PfCSP/Py parasite produced up to 30,000 Spz in mosquito salivary glands, which is equal or even higher than the number of Spz produced by wild-type *P. yoelii* parasites. Five bites of PfCSP/Py-infected mosquitoes could induce blood infection in BALB/c mice.

**Conclusions:**

The current study has demonstrated a successful establishment of a transgenic *P. yoelii* parasite clone that is able to express a full-length PfCSP, PfCSP/Py parasite. Importantly, this PfCSP/Py parasite can be as infectious as the wild-type *P. yoelii* parasite both in mosquito vector and in mouse, a mammalian host. A new transgenic parasite that expresses a full-length PfCSP may become a useful tool for researchers to investigate immunity against PfCSP in a mouse model.

## Background

There were 198 million cases of malaria (with an uncertainty range of 124–283 million) in 2013, which led to 584,000 fatalities (uncertainty range of 367,000–755,000) [1]. Malaria is transmitted among humans by female mosquitoes of the genus *Anopheles*. Sporozoites (Spz) are the infectious forms of malaria parasites residing in mosquito salivary glands [2, 3]. Radiation or genetically attenuated whole Spz vaccine candidates target the pre-erythrocytic stages in the life cycle of the parasite [4–6] and are shown to be highly protective, but they are challenging to mass manufacture and administer [2, 7, 8]. The circumsporozoite protein (CSP) is an immunodominant protective antigen present in malaria Spz [9, 10]. CSP is also a major component of the most advanced malaria vaccine candidate, RTS,S/AS01, also called Mosquirix^®^, which has been licensed for use in endemic countries by the European Medicines Agency [2]. A clinical phase III trial evaluated the vaccine efficacy against all episodes of severe malaria be approximately 50 % in young children in Africa [11, 12]. There was a correlation between antibody titres against *Plasmodium falciparum* CSP (PfCSP) and the degree of protection in both the RTS,S/AS01 vaccine and whole Spz vaccine, but the role of cell-mediated immunity in protection induced by the vaccines was not clear [13].

A recombinant rodent parasite, PfPb parasite, *Plasmodium berghei* bearing only the central repeat region of PfCSP, which is from the human parasite *P. falciparum*, was generated to study protective humoral immunity induced by the PfCSP central repeat [8, 14–16]. PfPb parasites were also used to investigate the in vivo protection induced by the anti-PfCSP monoclonal antibodies (mAbs) delivered by adeno-associated, virus-based gene transfer [17]. The lack of both N-terminal and C-terminal in the PfPb parasite is a drawback for its use in the analysis of protective immunity mediated by T-cells as the non-repeat regions contain the majority of the CD4+ and CD8+ T cell epitopes of PfCSP [18–20]. Recently, a recombinant *P. berghei* parasite expressing a full-length PfCSP was generated, but the parasite’s infectivity was very low in the mosquito salivary gland [21, 22].

Here, the current study describes the generation of a highly infectious, recombinant rodent malaria parasite, PfCSP/Py, a *Plasmodium yoelii* parasite expressing a full-length PfCSP, instead of *P. yoelii* CSP (PyCSP). This hybrid parasites’ level of infection in mosquito salivary gland is around 20,000–30,000/mosquito, and in vivo infectivity is equal to or higher than that of wild-type *P. yoelii*. This PfCSP/Py parasite provides malaria researchers with an effective tool to study the T-cell and antibody-mediated immune responses induced by a full-length PfCSP, as well as the protective efficacy of a full-length PfCSP-based vaccine in mice.

## Methods

### Mice

Six-week old female BALB/c mice were purchased from the Jackson Laboratory (Bar Harbor, ME, USA) and were maintained under standard conditions in the Laboratory Animal Research Center of The Rockefeller University. All animal experiments were carried out in strict accordance with the Policy on Humane Care and Use of Laboratory Animals of the US Public Health Service. The protocol was approved by the Institutional Animal Care and Use Committee (IACUC) at The Rockefeller University (Assurance # A3081-01).

### Construction of transgenic parasites

**Fig. 1:**
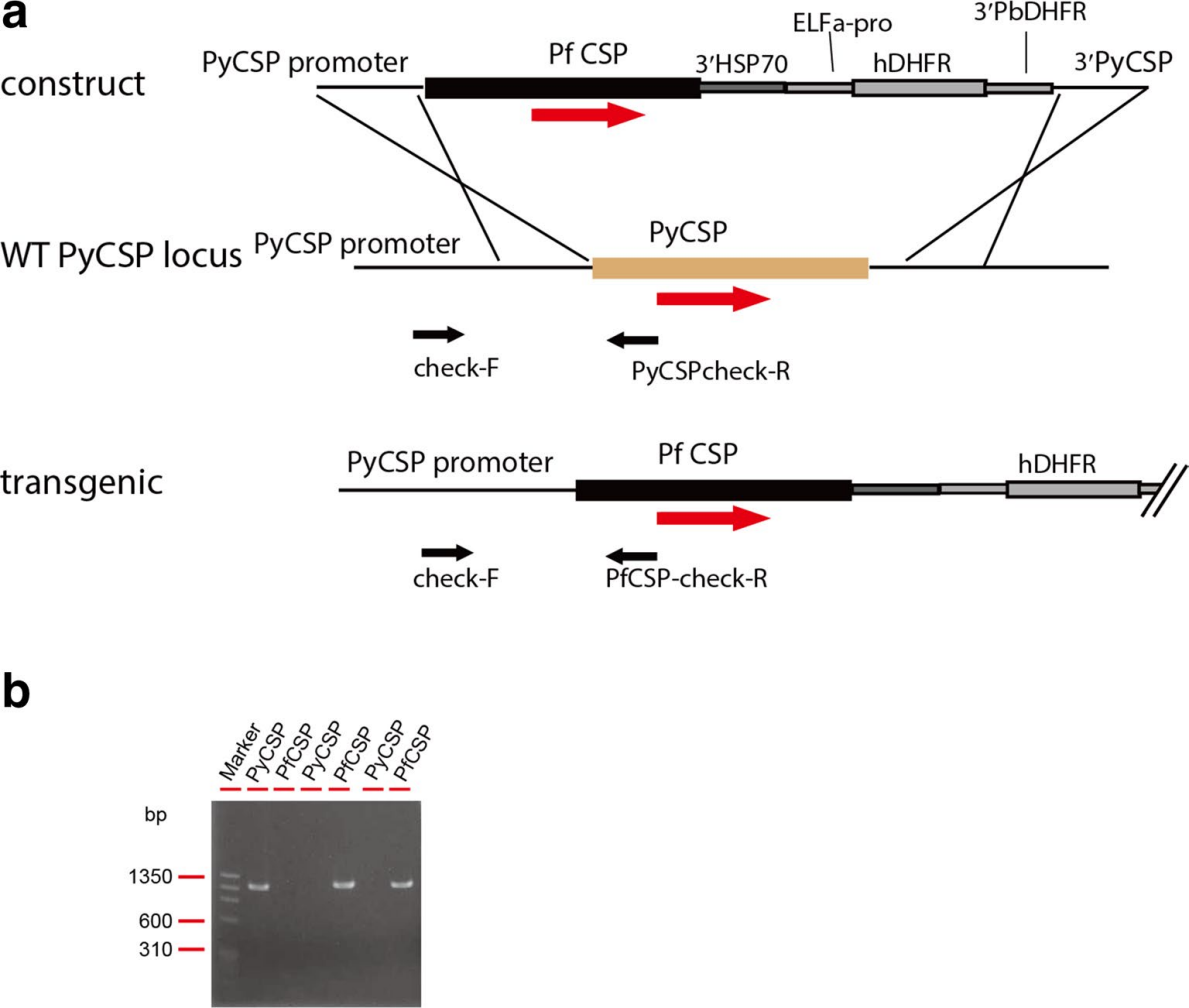
Generation of transgenic PfCSP/Py parasites. **a** Transgenic parasites were generated by inserting the PfCSP expression construct at the locus of the *P. yoelii CSP* gene by double cross-over homologous recombination. **b** Correct targeting was checked by gDNA PCR. Primers used in these experiments were listed in Table 1

Transgenic parasites were generated by inserting the PfCSP expression construct at the locus of the *P. yoelii CSP* gene by double cross-over homologous recombination (Fig. 1). The targeting plasmid for generating PfCSP/Py parasites was generated in a pBluescript plasmid that contains the PyCSP promoter, PfCSP coding sequence, 3′UTR of *HSP70*, human DHFR expression cassette, and 3′UTR of PyCSP. The plasmid was linearized by digestion with restriction enzymes KpnI and NotI. Transfection of *P. yoelii* 17XNL strain [23] was performed by the same procedures as described previously [24] except that a discontinuous gradient of Percoll/sorbitol (60/40 %) was used for purification of mature schizonts instead of Nycodentz. After transfection, parasites were selected by pyrimethamine in drinking water. Then, the resistant populations were single cloned by limiting dilution in mice. Correct targeting was checked by gDNA PCR. Primers used in these experiments were listed in Table 1.

**Table 1:** List of primers used for plasmid construction

Amplified region	Forward primer	Reverse primer
PyCSP promoter	CTATCAAATAATGTACTGCCCTTAAAAGC	GCTAATTTTCTCATCATTTTAAATATGTGTGTGTATATATAAG
PfCSP ORF	CATATTTAAAATGATGAGAAAATTAGCTATTTTATCTG	aaagtcgacGTTGTTCTTAATGATTCAATGCACGGTG
Three regions of PyCSP gene	aaaggatccGTATTGTGAACTTTCCTCATTTATTACG	aaagcggccgcCATATTTATGTACACCCTTTTGTGGACC
PyCSP primer forward	CTACGTAACAAATATGCAAGATGG	
PyCSP primer reverse		CATATCCTGGAAGTAGAGAATCAAC
PyCSP primer reverse		AGAACCCTTGTGTTTGACGAAC

### Measurement of the number of Spz in the mid-gut and salivary glands of mosquitoes

Approximately 300 female *Anopheles stephensi* mosquitoes were allowed to feed on a group of five Swiss-Webster mice infected with 0.1 % gametocyte of either wild-type *P. yoelii* 17XNL parasites or PfCSP/Py parasites, as previously described [25, 26]. Then, the midguts and salivary glands were dissected from a group of five infected mosquitoes from day 8 to day 26 post-infectious blood meal.

### Western-blot assay

The extracts of 1 × 10^4^ salivary gland Spz of PfCSP/Py, *P. falciparum* 3D7, or wild-type *P. yoelii* 17XNL were placed in sample buffer containing 2 % sodium dodecyl sulfate (SDS), 10 % glycerol, and 0.005 % bromophenol blue for 10 min. The extracts were then subjected to SDS–PAGE and electro-blotted onto PVDF membranes. The membranes were blocked by 5 % no-fat milk in TBST and incubated for 1 h with mAb, 2A10 specific for PfCSP repeats, or 2F6 specific for PyCSP repeats. After it was washed three times with Tris-buffer containing 0.05 % Tween-20, the membrane was incubated with goat anti-mouse IgG (H+L) antibody, which was detected using Pierce ECL western blotting substrate (Thermo Fisher Scientific Inc., Waltham, MA, USA).

### Immunofluorescence assay

Five× 10^3^ salivary gland Spz of PfCSP/Py or wild-type *P. yoelii* 17XNL were loaded onto MP Biomedical multi-test slides. After they were air dried at room temperature for 2 h, the slides were fixed with 4 % paraformaldehyde for 10 min, and then blocked with 3 % BSA in PBST. The slides were incubated with 2A10 for 45 min. After they were washed three times with PBS containing 0.05 % Tween-20, the slides were incubated with Alexa Fluor 594 goat anti-mouse IgG (H+L) antibody (Thermo Fisher Scientific Inc, Catalogue#: A-11005). After 1 h, the slides were washed and mounted in PBS containing 50 % glycerol and 1 % (w/v) p-phenylenediamine to reduce bleaching.

### Sporozoite infectivity

Infectivity of PfCSP/Py SPZ was determined in female BALB/c mice by two different methods. In the first method, mice were injected intravenously (iv) with 50, 150 and 450 Spz of PfCSP/Py or wild-type *P. yoelii* dissected from salivary glands. In the second method, mice were exposed to the bite(s) of one, five or ten PfCSP/Py parasite-infected mosquitoes. In both methods, the parasitaemia of the challenged mice was determined by microscopic examination of Giemsa-stained thin blood smears, obtained from days 3 to 10 post-Spz challenge.

### ELISA assay

Levels of antibody in mice immunized with PfCSP/Py immunized mice were determined by ELISA. Mouse sera collected before vaccination were used as a negative control. The ELISA plates were first coated with a recombinant full-length PfCSP (10 μg/ml) and then blocked with 3 % BSA in PBS-T. Mouse sera were twofold diluted (1:200–1:12,800) and added to the plates and incubated for 1 h. After washing the plates, HRP-conjugated goat anti-human IgG Fc Fragment was added. After TMB High Sensitivity Substrate was added, ODs were read at 450 nm. Mouse sera collected 1 day before challenge were analyzed for antibody titres against PfCSP.

### Mouse protection assay

Female BALB/c mice were immunized once or twice by iv injection with different doses of gamma-irradiated PfCSP/Py Spz, as described in Table 4. The interval between the priming and boosting was 14 days. Fourteen days after priming or boosting dose, mice were challenged by iv injection with 50 live PfCSP/Py Spz. Parasitaemia was determined by microscopic examination of Giemsa-stained thin blood smears, obtained at 7 days post-Spz challenge.

## Results

### Biological properties of PfCSP/Py parasites

**Fig. 2:**
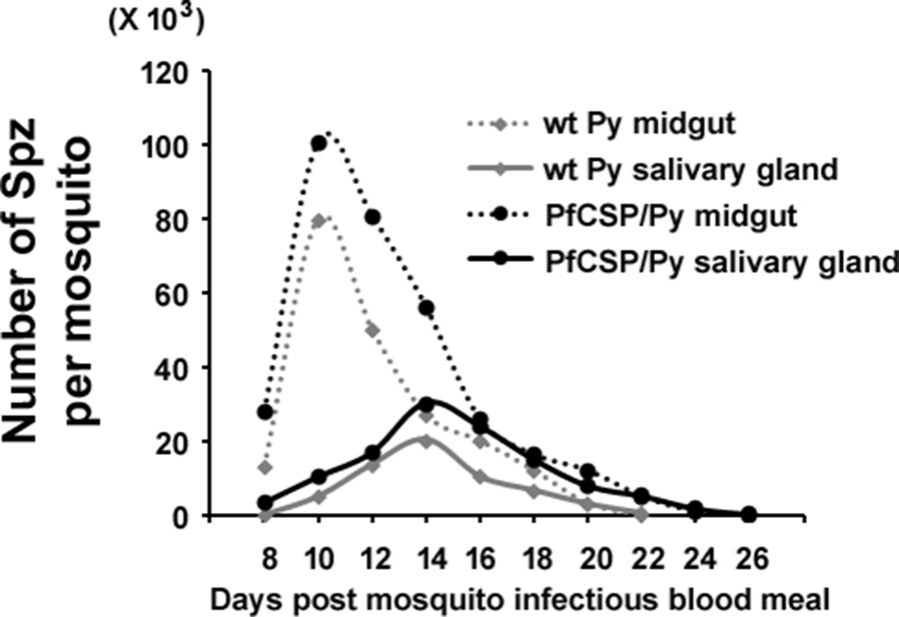
Hybrid PfCSP/Py parasites produced sporozoites in mosquito. Three-hundred female *Anopheles* mosquitoes fed on five Swiss-Webster mice infected with wild-type *P. yoelii* or transgenic PfCSP/Py parasites with 0.1 % gametocytaemia. Mosquito midguts and salivary glands from five mosquitoes were dissected from day 8 to day 26 post mosquito-infectious blood meal

A hybrid *P. yoelii* parasite has been generated by replacing full PyCSP with full PfCSP via double cross-over recombination. The full-length CSP is under the regulation of PyCSP untranslated regions (UTRs) (Fig. 1). The PfCSP/Py parasite completed the life cycle in *Anopheles* mosquitoes, and produced midgut and salivary gland Spz higher than or comparable to wild-type *P. yoelii* parasites (Fig. 2). For both PfCSP/Py and wild-type parasites, the date of highest number of midgut and salivary gland Spz post-infectious blood meal was around day 10 and day 14, respectively. The range of days with >10,000 salivary gland Spz per mosquito is 9 days long (day 10–day 18) for PfPy parasites, which is 5 days longer (day 12–day 16) than the wild-type counterpart.

**Fig. 3:**
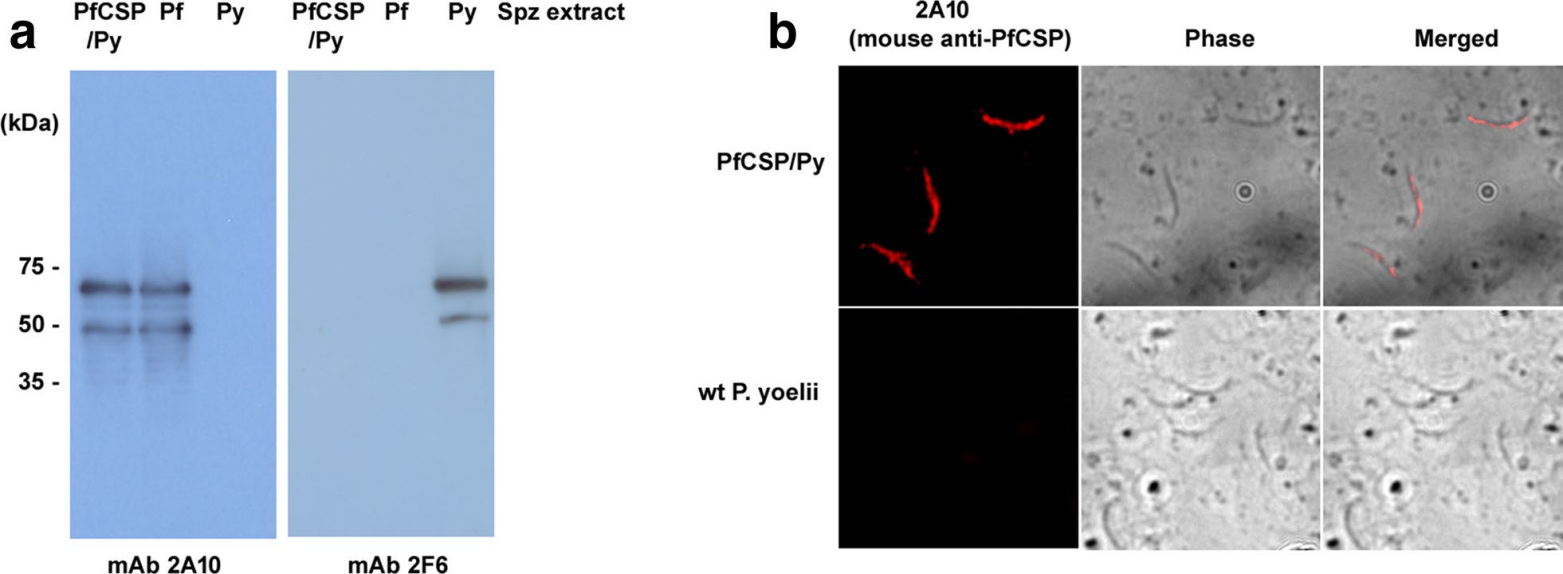
PfCSP/Py sporozoites (Spz) express *Plasmodium falciparum* CSP. **a** Immunoblots of PfCSP/Py, *P. falciparum* (Pf), and *P. yoelii* (Py) SPZ, using mAb 2A10 (recognizes PfCSP) and mAb 2F6 (recognizes PyCSP). The *bottom bands* represent processed products. **b** Staining of live Spz with mAb 2A10 that recognizes the central repeats of PfCSP

The hybrid PfCSP/Py Spz were recognized by 2A10 mAb, an antibody that specifically reacts with the central repeats of the PfCSP [27]. The 2F6 mAb, an antibody that reacts with the central repeats of the PyCSP [28, 29], had no reactivity to the hybrid PfCSP/Py Spz (Fig. 3). The immunoblot and immunofluorescent assay showed that the PfCSP/Py Spz expressed PfCSP, but not PyCSP.

### Titration of the PfCSP/Py Spz infectivity

BALB/c mice were injected intravenously with 50, 150 or 450 PfCSP/Py Spz or wild-type *P*. *yoelii* Spz, and monitored development of blood stage infection in the mice on days 3–5 post challenge. The PfCSP/Py Spz were highly infective, similar to wild-type *P. yoelii* Spz (Table 2). The appearance of erythrocytic stage parasites was observed 4 days post challenge in all of the mice injected with the low dose of PfCSP/Py Spz, whereas four out of five mice challenged with 50 wild-type *P. yoeli* Spz became positive.

**Table 2:** Titration of the infectivity of PfCSP/Py Spz through intravenous injection

	# of Spz injected iv	Day 3 post challenge^a^	Day 4 post challenge	Day 5 post challenge
Wild-type *P. yoelii*	450	0/5	5/5	5/5
	150	0/5	5/5	5/5
	50	0/5	4/5	5/5
PfCSP/Py	450	0/5	5/5	5/5
	150	0/5	5/5	5/5
	50	0/5	5/5	5/5

^a^Number of infected mice/number of challenged mice. Infection was defined as the observation of blood stage parasites in thin blood smears by Giemsa staining

Then the infectivity of PfCSP/Py Spz was evaluated in mice delivered by mosquito bite (Table 3). BALB/c mice were exposed to the bites of one, five or ten PfCSP/Py-infected mosquitoes. It was found that the bite of one PfCSP/Py-infected mosquito induced blood infection in 17 % (1/6) of mice, whereas five or ten bites of five or ten PfCSP/Py-infected mosquitoes induced blood infection in all (6/6) mice.

**Table 3:** Titration of the infectivity of PfCSP/Py Spz through the bite(s) of infected mosquitoes

	No. bites^a^	Infected^b^/total mice	% patent	PPP (days)^c^
BALB/c mice	1	1/6	17 %	5
	5	6/6	100 %	4.2
	10	6/6	100 %	3.8

^a^80 % of *An. stephensi* mosquito was infected with PfCSP/Py Spz in the salivary gland. Individual salivary glands were examined after each feed to ensure that mice received the indicated number of infected bites

^b^Infection was defined as the observation of blood stage parasites in thin blood smears by Giemsa staining

^c^Pre-patent period (PPP), number of days after Spz inoculation until detection of blood stage parasites in Giemsa-stained thin blood smears

### Protective efficacy of radiation-attenuated PfCSP/Py Spz

Then the protection of BALB/c mice following immunization with gamma-irradiated PfCSP/Py Spz was investigated. The mice immunized with one dose of 50,000, 100,000 or 200,000 irradiated PfCSP/Py Spz were all infected by day 7 post challenge with PfCSP/Py Spz. The mice immunized with two doses of 25,000, 50,000, or 100,000 PfCSP/Py Spz were protected in a dose-dependent manner against PfCSP/Py Spz challenge. The majority of mice (3/4) were protected in the group that received highest prime boost vaccination dose of 100,000/100,000 (Table 4).

**Table 4:** Protection of immunized BALB/c mice

	Vaccination/boost dose^a^	No. infected mice/no. challenged mice^b^
Irradiated PfCSP/Py	–	4/4
	200,000	4/4
	100,000	4/4
	50,000	4/4
	100,000/100,000	1/4
	50,000/50,000	2/4
	25,000/25,000	3/4

^a^The interval between the priming and boosting with irradiated PfCSP/Py Spz is 14 days

^b^Mice were challenged by iv injection of 50 live, non-irradiated salivary gland PfCSP/Py Spzs. The infection was evaluated by the presence of parasites in thin blood smears upon Giemsa staining on 7–14 days post Spz challenge

**Fig. 4:**
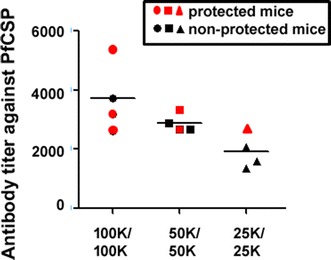
Antibody titres against PfCSP in the sera of PfCSP/Py-immunized mice. BALB/c mice were twice immunized with PfCSP/Py Spz. One day before challenge with live PfCSP/Py Spz, the sera were collected and the antibody titres were determined by ELISA. *Red* and *black symbols* represent the protected and non-protected mice (shown in Table 4), respectively

The titres of antibody against PfCSP induced in the sera of mice immunized with irradiated PfCSP/Py Spz were determined (Fig. 4). The sera were collected 1 day before challenge, or 13 days post boost. The CSP antibody titres of protected mice were higher than 2000, whereas the titres in mice that were not protected were lower than 2000 (Fig. 4). It is noteworthy that some mice that mounted high anti-CSP antibody titres were not protected, thus indicating that other immune responses may account for the overall protection.

## Discussion

The complete *P. falciparum* life cycle cannot be maintained in vitro in the laboratory, thus limiting the experimental study of the Spz of *P. falciparum* human malaria parasite. Laboratory mouse malaria models have provided alternative platforms for human malaria research. Rodent malaria (*P. yoelii*, *P. berghei*, *Plasmodium chabaudi*, and *Plasmodium vinckei*) isolated from wild thicket rats have been adapted to grow in laboratory rodents. These species display many of the biological characteristics of the human malaria parasite and rodent models of malaria have been widely and successfully used to complement research on *P. falciparum* [30, 31].

Although recombinant rodent *P. berghei* parasites expressing PfCSP repeats or a full-length PfCSP were generated, their infectivity in mosquito salivary glands was lower than that of wild-type parasites. Here, *P. yoelii* 17XNL was used [32], which produces high number of Spz in mosquito salivary glands and is infectious to mice of both BALB/c and C57BL/6 strains [33], as the parental line for generating the PfCSP/Py transgenic parasites. The CSP, the major antigen on the Spz surface, is composed of the N-terminal flanking region, the repetitive immunodominant B-cell epitope containing central region, T-cell epitope containing C-terminal flanking region. The N- terminal regions play essential roles in parasite infectivity and could be targeted by protective antibodies [34]. In this study, a transgenic *P. yoelii* parasite clone that is able to express a full-length PfCSP was successfully generated and its biological properties compared to those of wild-type *P. yoelii* parasite were investigated.

A novel clone of a transgenic *P. yoelii* parasite that is able to express a full-length PfCSP, named PfCSP/Py parasite, was generated. This PfCSP/Py parasite clone can produce up to 30,000 Spz in mosquito salivary gland, which are equal or even higher than the number of Spz produced by wild-type *P. yoelii* (Fig. 2). The number of days for collecting high numbers of salivary gland PfCSP/Py Spz (>10,000/mosquito) is around 9 days, which provides a wider range of working days for researchers than wild-type *P. yoelii*. When mice were exposed to the bites of PfCSP/Py-infected mosquitoes, it was found that bites of five PfCSP/Py-infected mosquitoes could induce blood infection in all of the mice (Table 3). This indicates that PfCSP/Py-infected *An. stephensi* mosquitoes can transmit malaria as efficiently as *P. yoelii*-infected *An. stephensi* mosquitoes, as demonstrated in a previous study [35]. The current study only compared the Spz numbers of PfCSP/Py parasites with those of wild-type *P. yoelii* through transmission from Swiss-Webster infected mice to *An. stephensi* mosquitoes. It is noteworthy that the parasites’ infectivity by transmission from other mouse strains, such as BALB/c and C57BL/6, to other mosquito species, such as *Anopheles freeborni* and *Anopheles gambiae*, requires further investigation.

The *P. falciparum* CSP has been regarded as one of the best vaccine candidates for malaria. Transgenic rodent malaria Spz expressing *P. falciparum* CSP represent a unique tool to evaluate PfCSP-based immunity and systematically assess formulation, molecular composition and regimen of a human malaria vaccine using a high-throughput animal experimental model. Although a recombinant rodent *P. berghei* parasite expressing PfCSP repeats was generated a decade ago, its infectivity in mosquito salivary glands has been modest, i.e., approximately 3000 per mosquito [8]. The infectivity of a more recently established, recombinant *P. berghei* parasite expressing a full-length PfCSP, is even less, i.e., only 1800 Spz are produced in the salivary gland [21]. The sequence changes introduced outside the conserved regions I and II by replacing PbCSP with PfCSP could possibly have reduced the ability of the PfCSP protein to function optimally within the *P. berghei* background during Spz invasion of *An. stephensi* salivary glands [21]. Thus, the yield of a low number of salivary gland Spz has probably hampered further PfCSP-based immunity and vaccines research, such as vaccination using irradiated PfCSP/Py Spz, which would require at least a million Spz per study.

Protective immunity induced by irradiated PfCSP/Py Spz has also been investigated. The protection rate of the mice that were immunized with the higher dose of vaccine (100,000/100,000) was much higher than that of mice that received the lower dose of vaccine (25,000/25,000). ELISA assay showed that there was a correlation between the titres of anti-PfCSP antibody and protection of the PfCSP/Py-vaccinated mice.

Lastly, when the titres of anti-PfCSP antibody titres were determined, there was a trend that mice that received a higher number of PfCSP/Py Spz produced a higher titre of anti-PfCSP. However, it is noteworthy that some mice that can mount higher anti-CSP antibody titres were not protected. This indicates that other immune responses may account for the overall protection.

Kumar et al. reported that the CSP is an immunodominant protective antigen in irradiated Spz [9], but Grüner et al. reported that sterile protection against malaria is independent of immune responses to the CSP [36]. In this regard, the authors are currently planning to conduct a comprehensive and independent study using heterologous immunization and challenge combinations with PfCSP/Py parasites and wild-type *P. yoelii* parasites in order to address this controversial issue.

## Conclusions

In summary, the current study has demonstrated a successful establishment of a transgenic *P. yoelii* parasite clone that is able to express the full-length PfCSP, PfCSP/Py parasite. Importantly, this PfCSP/Py parasite can be as infectious as, or even more than, wild-type *P. yoelii* parasite both in mosquito vector and in mouse, a mammalian host. This indicates that the expression of the PfCSP transgene does not interfere with the infectivity and fitness of the parasite. The new transgenic parasite that expresses a full-length PfCSP may become a useful tool for researchers to investigate immunity against PfCSP in a mouse model.

## Abbreviations

Spz: sporozoites
CSP: circumsporozoite protein
PfCSP: *P. falciparum* CSP
PfCSP/Py: *P. yoellii* expressing a full-length PfCSP
ELISA: enzyme-linked immunosorbent assay
mAb: monoclonal antibody
SDS: sodium dodecyl sulfate
